# Coronary Subclavian Steal Syndrome Causing Acute Myocardial Infarction in a Patient Undergoing Coronary-Artery Bypass Grafting

**DOI:** 10.1155/2012/798356

**Published:** 2012-08-30

**Authors:** Jiri Mandak, Miroslav Lojik, Martin Tuna, James Lago Chek

**Affiliations:** Department of Cardiac Surgery and Department of Radiology, Faculty of Medicine and University Hospital in Hradec Králové, Charles University in Prague, 50005 Hradec Králové, Czech Republic

## Abstract

Coronary subclavian steal syndrome with retrograde blood flow in the left internal mammary-coronary bypass graft is a rare but severe complication of cardiac surgery. The authors present a case of a 68-year-old man after coronary-artery bypass grafting using an internal mammary artery. He had been suffering from angina pectoris for the last several years before surgery. The patient was resuscitated at home by emergency medical service because of primary ventricular fibrillation due to an acute myocardial infarction 5 years after surgery. An occlusion of the left subclavian artery with the retrograde blood flow in the left internal mammary coronary bypass was found. This could have been the cause of insufficiency in coronary blood flow and ischemia of the myocardial muscle. The subclavian artery occlusion was successfully treated with percutaneous transluminal angioplasty and implantation of 2 stents. The patient remained free of any symptoms 2 years after this procedure.

## 1. Introduction

Coronary subclavian steal syndrome (CSSS) is a rare but potential risk entity after coronary surgery caused by retrograde blood flow through the internal mammary artery graft.

The internal mammary (internal thoracic) artery originates from the subclavian artery, and blood flow is dependent on inflow by the vessels in the aortic arch. Stenosis or occlusion of the subclavian artery may cause insufficient or retrograde blood flow in the mammary artery graft with steal phenomenon in the coronary bed. 

Angina pectoris, rated from moderate to unstable, ranks among the typical symptoms. Acute myocardial infarction (AMI), caused by this entity, is very rare. Only four reports were found in the literature [1–4]. 

## 2. A Case Report

The authors present a case of a 68-year-old man with ischemic heart disease (triple vessel disease, CCS III, EF 55%) who underwent on-pump myocardial revascularization—coronary artery bypass grafting (CABG) that included a left internal mammary artery (LIMA) graft to the left anterior descending branch (LAD) and vein grafts to the right coronary artery (RCA) and to the marginal branch of the left circumflex (LCx) branch. Blood flow through the LIMA was optimal after harvesting and before grafting with the LAD. The postoperative management was standard without complications. He had been suffering from angina pectoris for the last several years. 

The patient was operated on due to peripheral arterial occlusive disease before cardiac surgery. An aortofemoral bypass graft and endarterectomy of the left internal carotid artery and bypass of the right internal carotid artery were performed. No ischemic or neurological problems were described after these procedures in the following years. 

Unfortunately, no medical imaging of the aortic arch and its branches was performed before cardiac surgery.

The patient denied any symptoms of angina pectoris after coronary revascularization. He suddenly suffered from ventricular fibrillation and was resuscitated at home by emergency medical service because of primary ventricular fibrillation 5 years after cardiac surgery. An acute myocardial infarction of the anterior wall of the left ventricle was found as the cause of acute heart failure.

No information regarding physical activity or manual work in this patient prior to AIM could be ascertained.

**Figure 1:**
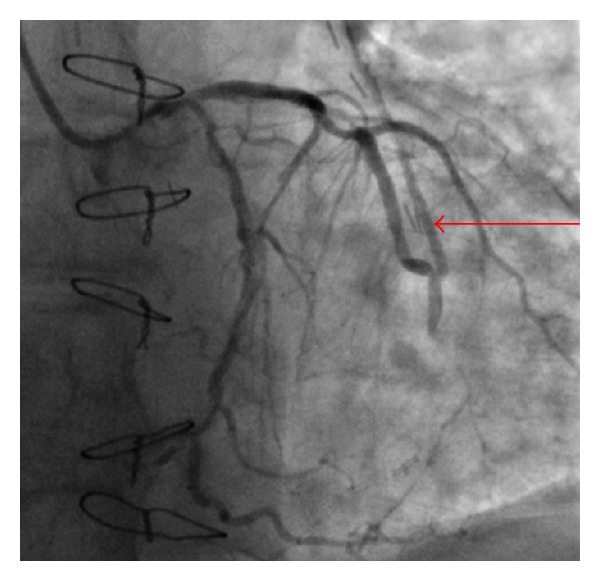
(a) Angiography of the left coronary artery. Optimal anastomosis of the LIMA to the LAD. Retrograde blood flow in the left mammary artery graft to the LAD—initial phase (arrow).

**Figure 2:**
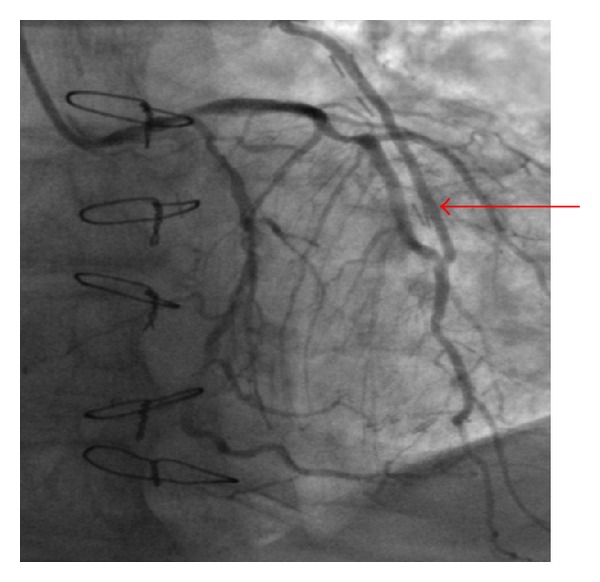
Angiography of the left coronary artery. Optimal anastomosis of the LIMA to the LAD. Retrograde blood flow in the left mammary artery graft to the LAD—later phase (arrow).

**Figure 3:**
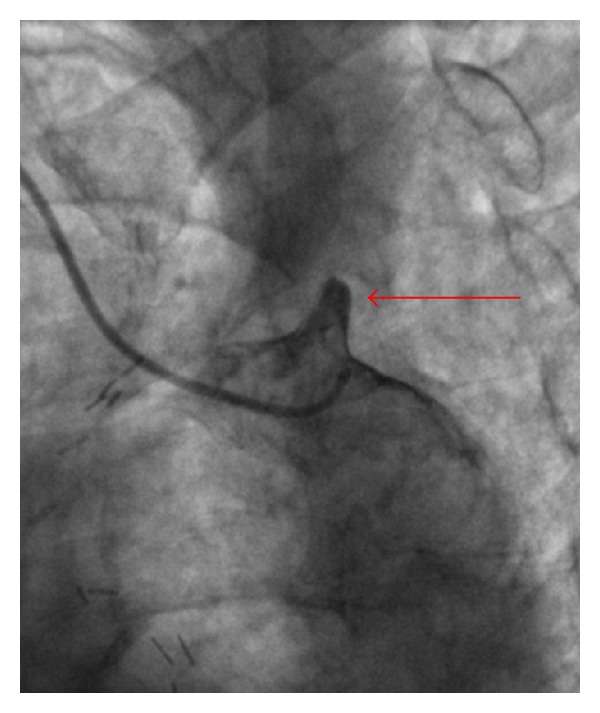
Selective angiogram of the origin of the occluded left subclavian artery (arrow).

**Figure 4:**
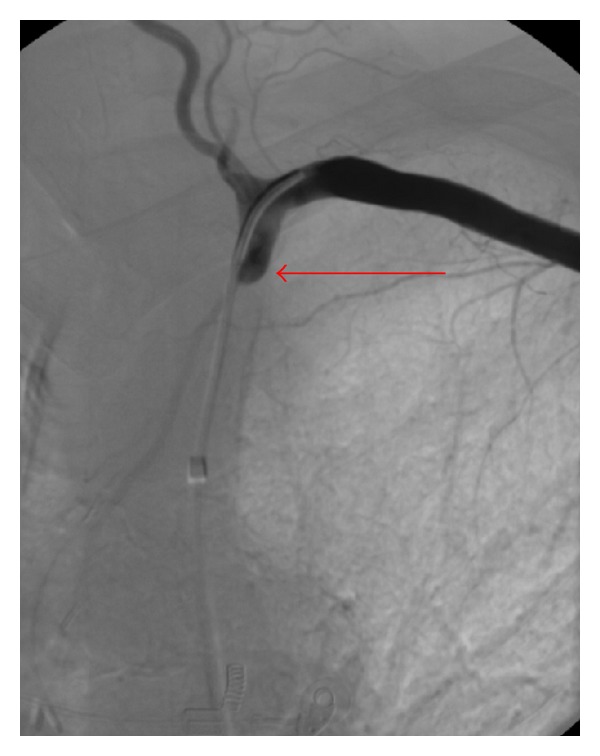
Angiography of the left subclavian artery after insertion of the guidewire (LIMA is not filled).

Coronary angiography revealed optimal anastomosis of the LIMA to the LAD, adequate blood flow through the vein graft to the LCx, and occluded bypass to the RCA with collateral blood supply from the left side. An occlusion of the left subclavian artery proximal to the LIMA origin with retrograde blood flow in the left internal mammary-coronary bypass was found (Figures 1–4). Probable reversed blood flow in the vertebral artery was not shown in this time. 

**Figure 5:**
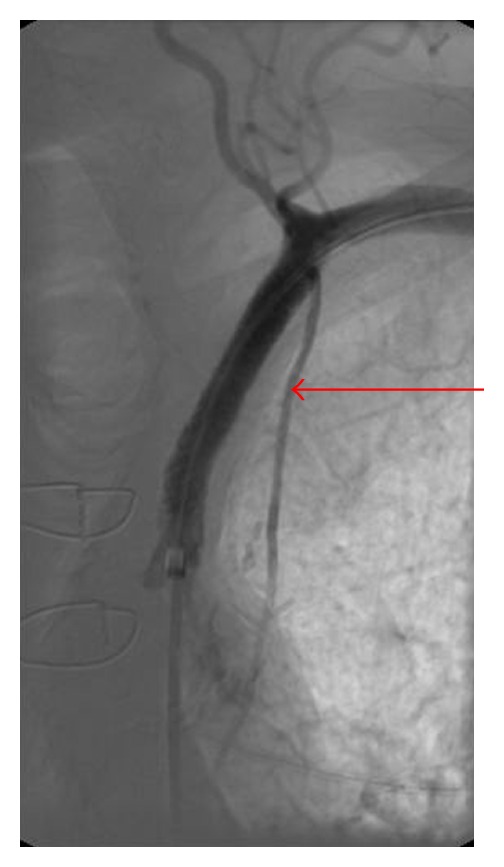
The left subclavian artery with implanted stents. Renewal of anterograde blood flow in the left mammary artery (arrows).

The subclavian artery occlusion was successfully treated with percutaneous transluminal angioplasty (PTA) via the right femoral artery (anticoagulation with 5000 units of intravenous heparin, sheath DAV 6 F, Terumo 0.035 inch diameter guidewire) and the implantation of 2 balloon expandable stents (Omnilink 8 × 27 mm, Omnilink 8 × 18 mm,) without any complications, 3 weeks after AMI (Figure 5). 

The patient was discharged 3 days after this procedure in good health. He has remained free of any symptoms 2 years after this procedure.

## 3. Discussion

Coronary subclavian steal syndrome with retrograde blood flow in the mammary-coronary bypass graft is a rare complication of cardiac surgery. 

The incidence of a symptomatic CSSS after coronary artery bypass surgery is low. Rossum et al. reported an incidence of 0.44% [5]. The prevalence of subclavian artery stenosis before cardiac surgery of 1.46% was presented by Van Noord [6].

Atherosclerosis is the most common cause of the steal syndrome (95%–97%) [1, 7]. Takayasu's arteritis, congenital aortic abnormalities, and thoracic outlet syndrome have also been described as possible causes in various reports [7–9].

The dominant symptom of the CSSS is angina pectoris, which depends on the degree of subclavian artery stenosis or occlusion and actual physical activity of the patient. Acute myocardial infarction is rare [1–4].

Clinically significant stenosis produces a differential forearm blood pressure of more than 20 mm Hg. A weak or absent radial and ulnar pulse in the presence of a reduced blood pressure when compared to the contralateral arm suggests insufficiency in subclavian blood flow. Left hand neurological or vascular symptoms (paresthesia, pain, cold, and pale-coloured skin) are not common [8].

Vertebrobasilar symptoms, typical for the subclavian steal syndrome, due to retrograde blood flow in the vertebral artery can be found too. This patient had no neurological problems.

Proximal aortic arch and direct subclavian artery arteriography are the gold standard for diagnosing subclavian diameter changes. Alternative diagnostic procedures are doppler, duplex ultrasonography, CT, or magnetic resonance [5, 10].

Treatment of choice in the case of stenosis or occlusion of the subclavian artery is percutaneous transluminal angioplasty with an intraluminal stent placement. If an interventional technique is not suitable for surgical revascularization by carotid-subclavian bypass or direct endarterectomy (with or without patch plasty) of the subclavian artery may be possible. Alternative treatment options focus on redo CABG, especially when other regions of the myocardium need to be revascularised. The LIMA could be used as a free graft. Of course a new vein graft to the LAD can be used. Coronary artery bypass reoperation can resolve only myocardial ischemia. Risk of other complications, which are done by occlusion of the subclavian artery, would still exist. 

We preferred PTA in this case due to a lower risk of the procedure than redo cardiac surgery. Aortic-subclavian bypass grafting, carotid-subclavian bypass grafting, or left subclavian endarterectomy are possible operations too, but a less invasive PTA is the method of the first choice. 

The cause of AMI in this case could have been due to occlusion of the subclavian artery. On the other hand, the occlusion by atherosclerotic disease is a chronic process. That is why it is likely that the cause of AMI could have been also due to insufficiency of the RCA graft which had been perfusing the LAD territory via collaterals.

The main point of discussion in this case was the absence of medical imaging of the aortic arch and its branches before cardiac surgery in 2004 when the previous vascular surgery was done. We had no information about the quality of the arterial bed of the patient. This knowledge could prevent possible future complications. Angiography of the aortic arch and its branches should be considered in patients with blood pressure difference between arms greater than 20 mm Hg prior CABG and in patients with some ischemic or neurological symptoms.

## 4. Conclusion

CSSS with retrograde blood flow in the mammary-coronary bypass graft causing acute myocardial infarction is a rare complication of cardiac surgery. Therapeutic approach must be individual, but we believe in PTA as the treatment of choice.
